# Demonstration of a positron beam-driven hollow channel plasma wakefield accelerator

**DOI:** 10.1038/ncomms11785

**Published:** 2016-06-02

**Authors:** Spencer Gessner, Erik Adli, James M. Allen, Weiming An, Christine I. Clarke, Chris E. Clayton, Sebastien Corde, J. P. Delahaye, Joel Frederico, Selina Z. Green, Carsten Hast, Mark J. Hogan, Chan Joshi, Carl A. Lindstrøm, Nate Lipkowitz, Michael Litos, Wei Lu, Kenneth A. Marsh, Warren B. Mori, Brendan O'Shea, Navid Vafaei-Najafabadi, Dieter Walz, Vitaly Yakimenko, Gerald Yocky

**Affiliations:** 1 SLAC National Accelerator Laboratory, Menlo Park, California 94025, USA; 2 Department of Physics, University of Oslo, 0316 Oslo, Norway; 3 Department of Electrical Engineering, University of California–Los Angeles, Los Angeles, California 90095, USA; 4 Department of Physics and Astronomy, University of California–Los Angeles, Los Angeles, California 90095, USA; 5 LOA, ENSTA ParisTech, CNRS, Ecole Polytechnique, Université Paris-Saclay, 91762 Palaiseau, France; 6 IFSA Collaborative Innovation Center, Department of Engineering Physics, Tsinghua University, Beijing 100084, China

## Abstract

Plasma wakefield accelerators have been used to accelerate electron and positron particle beams with gradients that are orders of magnitude larger than those achieved in conventional accelerators. In addition to being accelerated by the plasma wakefield, the beam particles also experience strong transverse forces that may disrupt the beam quality. Hollow plasma channels have been proposed as a technique for generating accelerating fields without transverse forces. Here we demonstrate a method for creating an extended hollow plasma channel and measure the wakefields created by an ultrarelativistic positron beam as it propagates through the channel. The plasma channel is created by directing a high-intensity laser pulse with a spatially modulated profile into lithium vapour, which results in an annular region of ionization. A peak decelerating field of 230 MeV m^−1^ is inferred from changes in the beam energy spectrum, in good agreement with theory and particle-in-cell simulations.

## Abstract (web summary)

Plasma wakefield accelerators produce gradients that are orders of magnitude larger than in conventional particle accelerator, but beams tend to be disrupted by transverse forces. Here the authors create an extended hollow plasma channel, which accelerates positrons without generating transverse forces.

## Graphical abstract

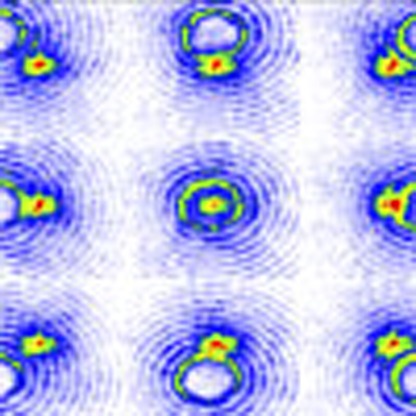

Plasma wakefield acceleration (PWFA) is a promising scheme for building compact and affordable accelerators^1^,^2^,^3^. Work on PWFA using uniform density plasmas has demonstrated acceleration of both electrons^4^ and positrons^5^ by collective fields in the plasma. Recent experiments have demonstrated high-gradient and highly efficient e^−^ and e^+^ acceleration in the extremely nonlinear regime of PWFA^6^,^7^. Wakes in the nonlinear regime have strong focusing forces in addition to strong accelerating fields that pose a challenge to preserving the extremely small emittance (phase space density) of the accelerating bunches^8^,^9^ that are required for high-energy collider and next-generation light source applications. A possible solution for preserving the emittance of beams accelerated in plasma is to use beam-induced wakes in a hollow channel surrounded by an annular plasma^10^,^11^,^12^,^13^. When an electron or positron bunch propagates on-axis through a hollow plasma channel, it induces a wakefield within the channel. The wakefield, moving synchronously with the beam, has an oscillating longitudinal field that is uniform in the transverse direction and a transverse force that is zero everywhere. Therefore, an appropriately placed trailing bunch of positive or negative charge can be accelerated without an increase in the emittance of the bunch. In spite of this critical advantage, there is little experimental work on wakefield excitation in hollow channel plasmas^14^.

Here, we produce and diagnose 8-cm long, hollow plasma channels and measure the decelerating phase of the longitudinal field excited by a positron bunch as it propagates through the channel. We find that when the positron beam propagates on-axis through the channel, there are no significant changes in the spatial profile, indicating the absence of the transverse focusing forces within the channel, thus demonstrating the merit of using hollow channel plasmas for PWFA.

## Results

### Experimental layout and positron beam parameters

**Figure 1:**
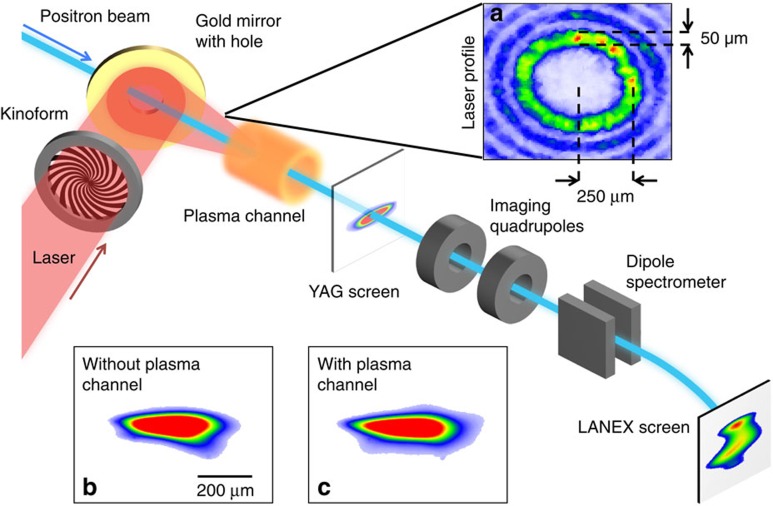
Experimental layout. The laser passes through the kinoform and is coupled to the beam axis by a gold mirror with a small central hole. Inset (**a**) shows the laser profile upstream of the lithium oven. A scintillating YAG screen 1.95 m downstream of plasma is used to measure the positron beam profile. Inset (**b**) shows the positron beam spatial profile as imaged on the YAG screen with the laser off and no plasma present. Inset (**c**) shows the beam profile with the laser on when the positron beam propagates through the plasma channel. The two profiles are similar, indicating that there are no net focusing forces because of the plasma channel. A scintillating Lanex screen downstream of the dipole measures the beam energy spectrum.

The experiment was carried out at the Facility for Advanced aCcelerator Experimental Tests (FACET) facility at the SLAC National Accelerator Laboratory^15^. The hollow plasma channel is formed when a high-intensity laser with a high-order Bessel profile field-ionizes a lithium vapour with number density *n*_0_=8 × 10^16^ cm^−3^. The lithium vapour is confined within a 130-cm-long heat-pipe oven at equal pressure with a helium buffer gas^16^. The experimental setup is depicted in Fig. 1.

The FACET Ti:sapphire laser^17^ delivered 100 fs FWHM laser pulses with 34 mJ per pulse to the lithium oven. The laser acquires a high-order Bessel profile by passing through a kinoform, which imprints a phase pattern on the laser (see the Methods for details). The laser is focused close to the downstream end of the Li column resulting in an 8-cm-long hollow plasma channel. The laser pulse is synchronized with the arrival time of the positron beam such that the laser ionizes the channel 3±0.25 ps before the beam arrives. The field ionization process produces a cold plasma where the liberated electrons have about an eV of energy. Therefore, the plasma diffusion into the channel can be neglected^18^. We note that if the Bessel beam is intense enough, it can additionally drive its own wake^19^.

The positron beam has a mean energy of 20.35 GeV with 350 MeV energy spread (FWHM). Lithium is readily ionized by the electric fields of intense particle beams^4^, so it is necessary to keep the particle beam density low enough that this does not occur. The particle beam is brought to a focus near the start of the plasma channel with 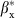 and 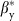 of 50 and 75 cm, respectively, where β*=*σ*^2^/*ɛ* is the divergence length of the particle beam, and *ɛ* is the beam emittance. The plasma channel is much shorter than β*, so the beam size is nearly constant over the length of the channel, with *σ*_x_≈*σ*_y_≈50 μm. Note that in the absence of plasma focusing forces, the length of the channel for a well-centred beam is determined by the beam emittance and divergence length, such that 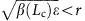, where *r* is the radius of the channel and *β*(*L*_c_) is the value of the *β*-function at the end of a channel with length *L*_c_. There are *N*=5.34 × 10^9^ positrons per bunch with an root mean square (r.m.s.) bunch length of *σ*_z_=35±5 μm, corresponding to a 2.9-kA peak current and a peak electric field of 1.6 GV m^−1^, well below the 7 GV m^−1^ field ionization threshold for lithium^20^.

### Modal description of the plasma wakefield

**Figure 2:**
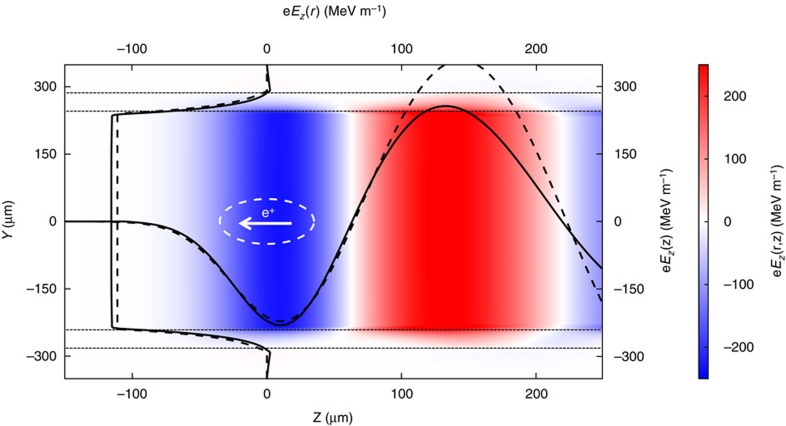
Simulation of the longitudinal field. The *E*_z_ field due to an ultrarelativistic positron beam driving a wake in a hollow channel plasma, as simulated by QuickPIC^34^,^35^. The beam propagates to the left and the 1*σ* contour of the beam is shown by the white dashed line. The black dotted lines at ±240 and ±290 μm are the inner and outer radii of the plasma channel, respectively. Lineouts of the simulated and calculated on-axis *E*_z_ field at *r*=0 μm and the radial variation in *E*_z_ at the peak decelerating field at *z*=11 μm are shown with solid and dashed black lines, respectively. The simulated and calculated fields show excellent agreement up to *z*=80 μm, where the charge separation of the plasma electrons on the surface of the channel becomes significant.

The beam excites electromagnetic modes as it propagates through the plasma channel. Following the procedure described in ref. 12, we calculate the wavelength, amplitude and single-particle wakefunction of the *m*=0, TM mode in a hollow plasma channel (see the Methods for details), and compute the longitudinal field experienced by the positron beam by convolving the wakefunction with the beam charge distribution. We compare our calculation to the results of a three-dimensional particle-in-cell simulation shown in Fig. 2 using the experimental parameters stated above. The maximum decelerating gradient experienced by the beam is ∼220 MeV m^−1^. The results of the calculation (dashed line) are well matched to simulation (solid line) up to a longitudinal value of *z*=80 μm, where the charge separation of the plasma electrons on the surface of the channel becomes significant (see the Methods for details).

We use the same analytical framework to calculate transverse forces experienced by the beam if it propagates off-axis through the plasma channel. In particular, we are interested in the fields associated with the *m*=1 dipole mode, which is the strongest mode contributing to the transverse beam break up instability^21^. The growth length for this instability is derived under the assumption that the change in beam energy is small over the length of channel^22^. The growth length for the beam centroid is given by


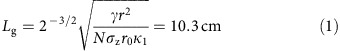


with beam energy *γ*, inner channel radius *r*, beam charge *N*, bunch length *σ*_z_, classical electron radius *r*_0_ and loss factor *κ*_1_. The growth length is comparable to the length of the plasma channel created in the experiment. The drift trajectory of the beam centroid is given by


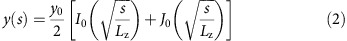


where *s* is the propagation distance, *y*_0_ is the initial transverse offset and *I*_0_ and *J*_0_ are zeroth order Bessel functions. A similar expression proportional to *I*_1_ and *J*_1_ can be derived for a beam with an initial angular offset. Note that the growth of this instability is relatively slow. For an initial offset of 5 μm (the r.m.s. beam orbit jitter), the beam can propagate for over a metre in the channel before the bunch reaches the channel wall.

### Characterization of the plasma channel

In general, the excitation of high-order modes in the channel will have deleterious effects on the beam. However, we can use the transverse fields associated with these modes to characterize the shape of the hollow channel plasma. The direction of the net transverse force on the beam points towards the nearest ionized region. For example, if the beam is offset vertically above the channel axis, it will be kicked towards the top of the channel. If the beam is outside the channel wall, it will experience a kick towards the nearest part of the plasma annulus. We thus can perform a raster scan of the laser with respect to the beam trajectory to map out the topology of the plasma channel.

**Figure 3:**
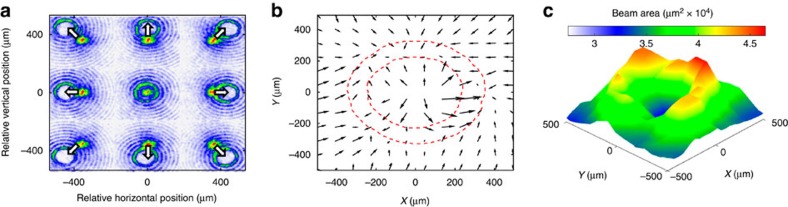
Determining the shape of the plasma channel. (**a**) A mosaic of images illustrating the principle behind the raster scan. Each subimage shows both the positron beam (with central hot spot) and laser profile (with ring) at low intensity reflecting off of a titanium foil. The laser is scanned in the transverse plane while remaining parallel to the beam trajectory. The arrows indicate the direction of force that the positron beam experiences for a plasma channel located at the position of the laser. (**b**) The kick map shows the magnitude and direction of the kick delivered to the beam averaged over ∼10 shots as the channel location is scanned with respect to the beam trajectory. A net kick of (43.6,48.7) μrad in (*x*,*y*) is subtracted from the data. We superimpose the intensity contour of the central Bessel peak of the laser measured upstream of the plasma as a red dashed line. (**c**) Area of the positron beam measured on a YAG screen downstream of the plasma averaged over ∼10 shots as the channel location is scanned with respect to the beam trajectory.

The raster scan is performed by first aligning the laser to the beam trajectory in a bypass line parallel to the lithium oven. There are two metallic optical transition radiation foils in the bypass line that are situated upstream and downstream of the lithium oven and separated by 1.84 m (see Supplementary Fig. 1 for details). Optical transition radiation light is produced by the positron beam passing through the foil and attenuated laser light is reflected from the foil, allowing the beam and laser position to be imaged simultaneously, as shown in Fig. 3a. The laser trajectory is set by the position of the kinoform, which is mounted on a stage that can be actuated horizontally and vertically, and by a gold folding mirror with tip-tilt action for a total of four degrees of freedom that are exploited to simultaneously align the beam and laser at the upstream and downstream foil locations. The angular alignment accuracy of the laser is limited by the pointing jitter of the laser, which was measured to be 25.4 μrad in *x* and 12.8 μrad in *y* r.m.s. With the laser aligned to the beam, the lithium oven is translated into place and the laser intensity is increased to ionize the vapour. We use the kinoform stage to raster the laser in the transverse plane while keeping the pointing of the laser parallel to the beam. The ionized plasma delivers a kick to the beam, which is recorded on a scintillating YAG screen 1.95 m downstream of the interaction region.

We observed an average kick in the +*x*, +*y* direction of 43.6 and 48.7 μrad, respectively, during the scan. We believe that this is due to an alignment error that occurred when the lithium heat-pipe oven was actuated into the beamline. The vector field, or ‘kick map', in Fig. 3b shows the net kick to the beam after subtracting off the average kick. In the experiment, the location of the laser is varied with respect to the beam trajectory, but for clarity we plot the kick vectors as though the laser is fixed and the beam trajectory is changed.

The shape of the kick map is consistent with our expectations for an annular ionized region. When the beam is outside the channel, the kicks point radially inwards towards the ionized region. When the beam is inside the channel, the kicks point radially outward toward the walls of the channel. The radial direction of the kick reverses sign in an annular region at a radius of 250 μm. Figure 3c shows the change in the beam area as measured on the same YAG screen. The transverse wakefield increases in strength longitudinally throughout the bunch. Particles towards the back of the bunch see a larger kick than particles in the front. The beam profile stretches in the direction of the kick, and the beam area increases as a result. The largest beam area growth occurs when the beam propagates along or through the ionized annulus. Both the kick map and beam area map show an annular feature with some asymmetry, which may be attributed to variations in the laser intensity around the first maximum of the Bessel profile, resulting in uneven ionization of the plasma annulus (see the Methods for details).

### Energy loss measurements

With the beam aligned to the centre of the channel, we can study the peak magnitude of the decelerating longitudinal field of the wake by measuring changes to the beam energy. The FACET energy spectrometer^23^ is comprised of an imaging quadrupole doublet and a vertically deflecting dipole magnet (see the Methods for details). The total systematic uncertainty in the energy measurement, including orbital effects, is 3.6 MeV (ref. 24).

**Figure 4:**
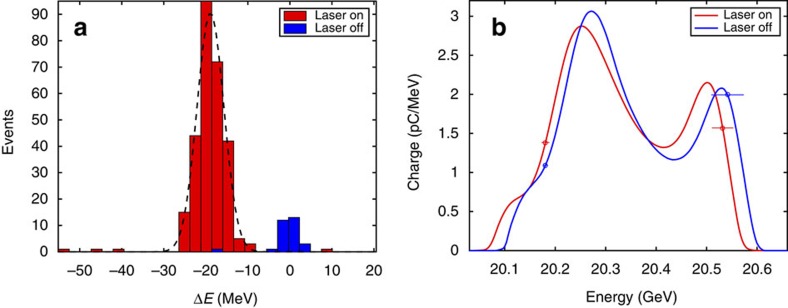
Energy loss measurements. (**a**) A histogram of the beam energy loss for all 315 shots corrected for incoming energy jitter (see the Methods for details). The plasma channel is present when the laser is on (red). When the laser is off (blue) the beam is propagating through neutral lithium vapour. We fit the laser-on data to a gaussian (black dashed curve) with mean energy loss 18.9 MeV and width 3.2 MeV. (**b**) A comparison of the average beam energy spectra for laser on and laser off shots. The s.d. error bars represent the statistical uncertainty in the upper and lower regions of the spectrum due to averaging (see Supplementary Fig. 4 for details). The error has been multiplied by a factor of five so that it is visible in the plot.

Figure 4a shows a histogram of the measured energy loss for a data set with 315 shots, 10% of which were taken with the laser off and therefore no plasma channel present. We measure a mean shift in the centroid beam energy of 18.9±3.20(stat)±3.55(syst) MeV. Figure 4b shows averaged energy spectra for the laser on and off cases.

To determine the gradient of the hollow channel plasma wakefield, we need an estimate of the channel length. This is achieved by measuring the transverse kick to the beam for small offsets in the channel, and comparing our result to the derivative of equation 2 evaluated at *s*=*L*_c_, where *L*_c_ is the length of the channel. The measured kick relative to the incoming beam orbit is 0.25 μrad per μm offset, corresponding to a channel length of 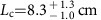, where the dominant contribution to the uncertainty comes from the uncertainty in bunch length. Note that the beam drift due to an initial angle 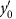 also contributes to the kick felt by the beam, but can be neglected if 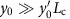, as is the case here. Therefore, the gradient we measure is 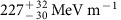. This is in excellent agreement with the peak decelerating field of 220 MeV m^−1^ shown in Fig. 2 from theory and simulations.

## Discussion

In the field of laser wakefield acceleration, the use of shallow plasma channels for laser guiding has become a common technique for extending the interaction length^25^. Our work is different in that we use a channel that has a centre region devoid of plasma, up to 240 μm in radius, surrounded by a plasma ring. One critical aspect of our experiment is to ensure that the effects we have measured are not due to residual, on-axis, low-density plasma. In selecting the beam parameters for our experiment, we reduced the number of beam particles from its nominal value of 2 × 10^10^ particles per bunch, while keeping the beam size constant, and sending the beam through the lithium oven with the laser off. We observed the beam profile and energy spectrum and found the threshold for interaction to be 1 × 10^10^ particles per bunch. The number of beam particles was further reduced to 5 × 10^9^, at which point there is no discernible difference in the beam profile or energy spectrum with and without the lithium oven.

The measured energy change has a distinct on–off effect related to the presence of the laser with the beam passing through the lithium oven. Profile measurements of the laser show better than 60:1 contrast between the intensity in the annular ring and the centre region of the Bessel profile. The Ammosov-Delone-Krainov (ADK) ionization rate^26^ for laser light below this intensity level is far to small to ionize lithium on-axis. Second, as shown in the inset of Fig.1, the positron beam profile with and without the laser produced plasma channel is nearly unchanged when the beam propagates through the centre of the channel. Previous work on positron beam propagation through uniform low-density (*n*_0_≈10^13^ cm^−3^) plasma columns has shown the formation of a halo of charge surrounding a central intense spot due to transversely nonlinear focusing forces acting on different slices of the beam^27^,^28^. No such halo formation or the formation of a central hot spot was observed here. This shows that there are no measurable focusing forces on the positron bunch, as expected from propagation in a hollow plasma channel. Finally, we note that the inferred gradient of 230 MeV m^−1^ is in excellent agreement with both our theoretical prediction and simulation result of 220 MeV m^−1^, as seen in Fig. 2.

In conclusion, this work demonstrates the core techniques for generating and probing hollow channel plasmas and shows that wakefields can be excited in such channels by the passage of a positron beam. The technique can be straightforwardly applied to produce metre-scale hollow channel plasmas, and the gradient can be increased by reducing the diameter of the channel and increasing the beam charge. Future experiments will explore the accelerating phase of the wake using an appropriately placed trailing bunch^6^. With even lower emittance beams, it may be possible to explore the non-linear regime of hollow channel PWFA, where the size of the channel is on the order of a plasma skin depth^29^,^30^,^31^. Such accelerating structures have no transverse focusing forces and therefore might be extremely attractive for stacking multiple accelerator stages while minimizing the emittance growth of the accelerating beam.

## Methods

### Plasma generation

The plasma channel is created by ionizing neutral lithium vapour with a high-intensity laser pulse that is shaped into a high-order Bessel profile using a kinoform phase plate. The kinoform is a 1-mm-thick piece of fused silica with an etched pattern that approximates the ideal phase Φ=*k*_⊥_*r*+*mϕ* to give a high-order Bessel profile, where *r* and ϕ are the radial and azimuthal coordinates, *m* is the Bessel order and *k*_⊥_=*k* sin(*α*) with *α* the angle of focused rays with respect to the axis and *k* is the wavenumber for 800 nm light^13^,^32^,^33^. We chose *m*=7 and *α*=4.4 mrad, which produces a *J*_7_ profile with the first maximum occurring at a radius of 250 μm. The resulting laser intensity is given by *I*(*r*, *z*)=*ηI*_0_2*πkz* sin(*α*)^2^*J*
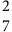
(*k*_⊥_*r*), with *η* the first-order diffraction efficiency, measured to be 45%. Here, *I*_0_=2.7 × 10^10^ W cm^−2^ is the incident laser intensity.

### Bessel profile optimization

The FACET Ti:Sapphire laser system contains a relay imaging system that is used to transport and telescope the laser over long distances. The lenses used in this system are potential sources of astigmatism. At the start of the experiment, these lenses are adjusted to remove any observed astigmatism in the first Bessel profile (see Supplementary Fig. 2a,b for details). The profile may also be uneven as a result of partial illumination of the kinoform optic (see Supplementary Fig. 2c,d for details). This is corrected by centring the laser on the kinoform. Note that the fully amplified laser may have a different intensity profile than the attenuated laser, which is used in the optimization procedure. An uneven intensity profile may lead to uneven ionization of the plasma near the first Bessel maximum, which a potential source of asymmetry observed in the kick map.

### Calculation of the longitudinal electric field

The longitudinal electric field experienced by the positron-drive beam is the convolution of the bunch charge distribution with the single-particle wakefunction. The wakefunction is computed by solving the electromagnetic wave equation inside the channel, in the plasma annulus, and outside the channel and by matching the boundary conditions. The plasma is represented by the dielectric function 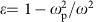 and is confined to the region *a*<*r*<*b*, where *a* and *b* are the inner and outer radii of the plasma annulus, respectively. We assume that the plasma electrons are not significantly displaced from their initial positions by the passing field of the beam. This assumption holds for a low-charge drive beam, as was the case in the experiment.

The wave equation is sourced by an on-axis, point charge driver propagating through the plasma channel. Only the co-propagating mode with *v*=*c* is considered, and the resulting single-particle wakefunction is given by


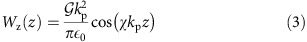


with *k*_p_ the plasma wavenumber. 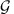 and *χ* are geometric quantities related to the wake amplitude and wavelength, respectively, and are determined by the inner radius and outer radius of the plasma annulus


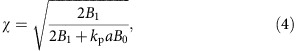



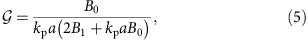


with


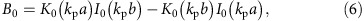



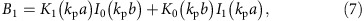


where *I*_n_ and *K*_n_ are the modified Bessel functions.

The drive beam is assumed to be Gaussian with charge distribution given by


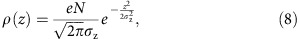


where *N* is the number of beam particles and *σ*_z_ is the bunch length. The longitudinal electric field experienced by the beam is the convolution of the wakefunction and charge distribution


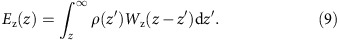


For the experimental parameters stated in the paper, we compute the maximum decelerating field to be 222 MV m^−1^.

### Imaging energy spectrometer

The beam energy is measured with an imaging spectrometer composed of a quadrupole doublet and vertical bend magnet downstream of the interaction region. The quadrupoles are set to image the beam waist, located at the start of the plasma channel, to a phosphorescent (LANEX) screen located 9.6 m downstream of the spectrometer dipole. The incoming beam energy from the linac has an r.m.s. jitter of 18.1 MeV, but we use a non-destructive energy spectrometer in the FACET chicane upstream of the interaction region to measure and correct for energy changes on a shot-by-shot basis (see Supplementary Fig. 3 for details). The energy resolution of the spectrometer is 3.1 MeV. Additional systematic errors in the energy measurement may arise from orbital effects. In particular, we checked for correlations in energy loss and vertical kicks from the plasma channel, but none were observed. This is consistent with the lattice imaging condition we used, which limited *R*_34_ to 0.11 m. The observed kicks from the plasma have a maximum amplitude of 30 μrad, producing a vertical displacement of 3.3 μm, or ∼1 MeV at the LANEX screen, which is too small to measure.

### Data availability

The data that support the findings of this study are available from the corresponding author upon request.

## Additional information

**How to cite this article:** Gessner, S. *et al*. Demonstration of a positron beam-driven hollow channel plasma wakefield accelerator. *Nat. Commun.* 7:11785 doi: 10.1038/ncomms11785 (2016).

## Supplementary Material

Supplementary Information Supplementary Figures 1-4
