# Supplementary material for: Demonstration of a positron beam-driven hollow channel plasma wakefield accelerator
Source: Nat Commun. 2016 Jun 2;7:11785. doi: 10.1038/ncomms11785 (PMC4895722; doi:10.1038/ncomms11785)
Supplement: Supplementary Information — Supplementary Figures 1-4 [file ncomms11785-s1.pdf]

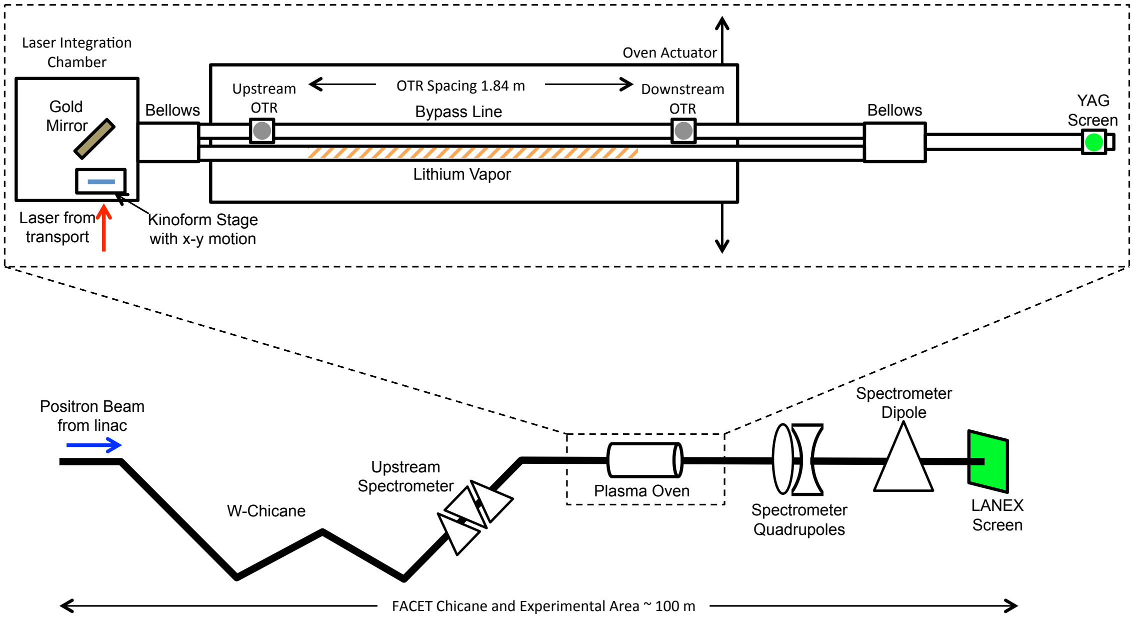

Supplementary Figure 1. Line drawing of experiment at FACET detailing all components discussed in the article. The W-chicane compresses the positron beam to its final bunch length of 35 microns. The inset is to scale.

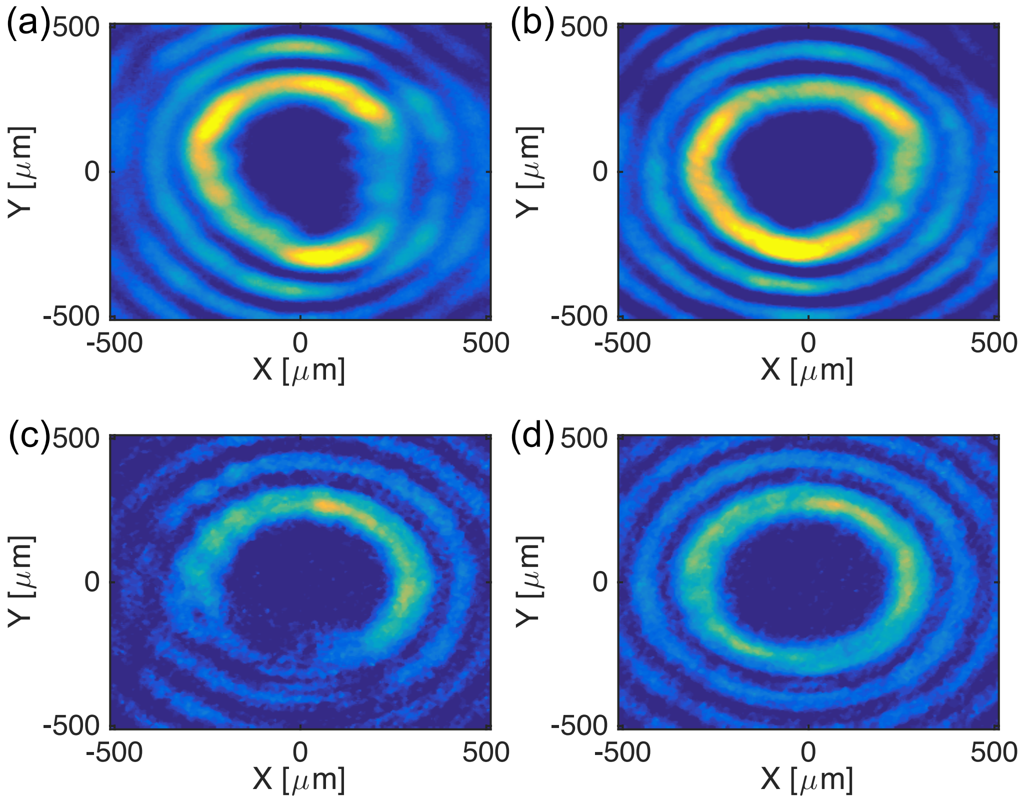

Supplementary Figure 2. Laser profiles before and after correction. (a) Uncorrected and (b) corrected astigmatism of the Bessel profile. (c) Uncorrected and (d) corrected illumination of the kinoform optic.

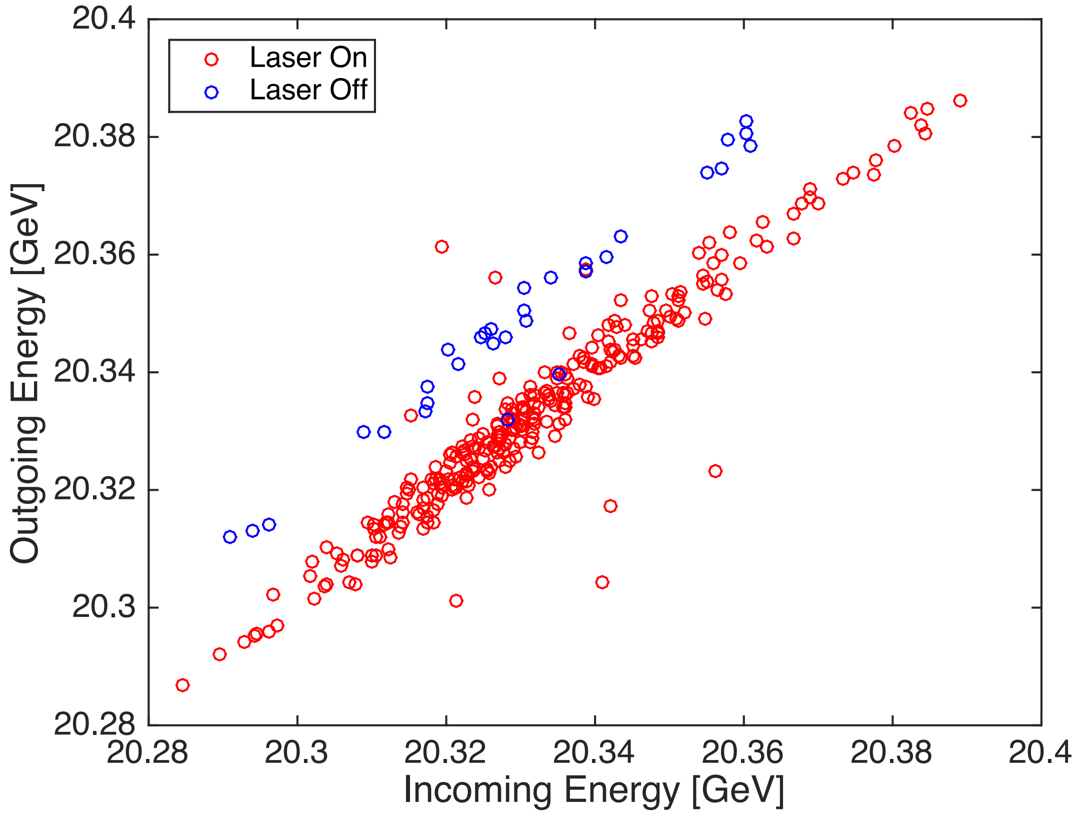

Supplementary Figure 3. Incoming energy, measured at the upstream spectrometer, versus outgoing energy, measured at the downstream spectrometer after the plasma channel for laser on and off shots.

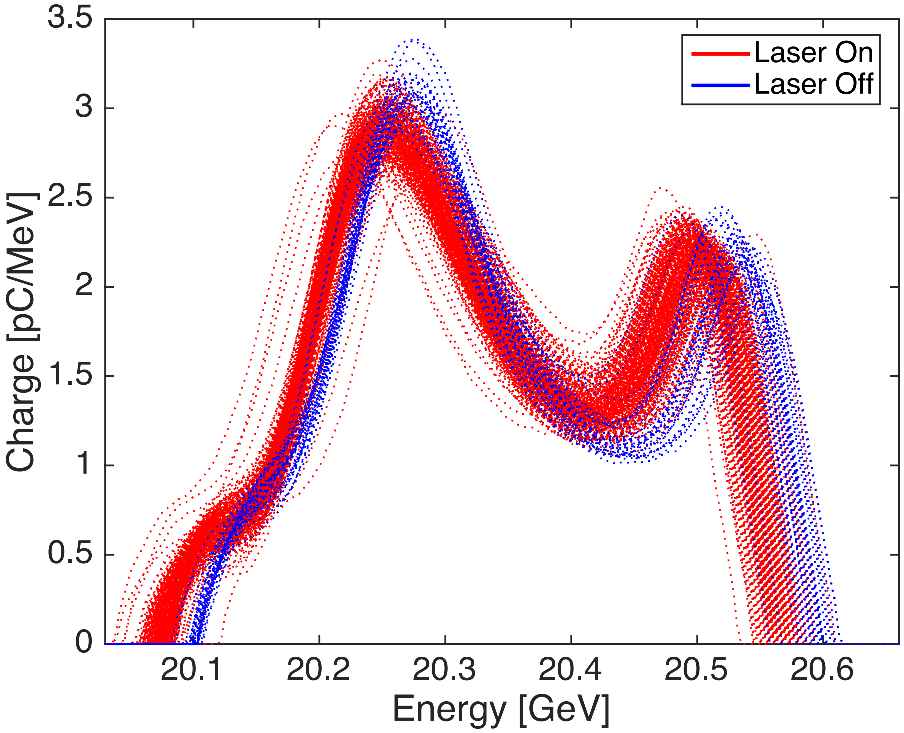

Supplementary Figure 4. Positron beam energy spectra corrected for incoming energy jitter for all shots in the dataset.
